# The Leukotriene B_4_/BLT_1_ Axis Is a Key Determinant in Susceptibility and Resistance to Histoplasmosis

**DOI:** 10.1371/journal.pone.0085083

**Published:** 2014-01-21

**Authors:** Adriana Secatto, Elyara Maria Soares, Gisele Aparecida Locachevic, Patricia Aparecida Assis, Francisco Wanderlei Garcia Paula-Silva, Carlos Henrique Serezani, Alexandra Ivo de Medeiros, Lúcia Helena Faccioli

**Affiliations:** 1 Departamento de Análises Clínicas, Toxicológicas e Bromatológicas, Faculdade de Ciências Farmacêuticas de Ribeirão Preto, Universidade de São Paulo, Ribeirão Preto, São Paulo, Brazil; 2 Departamento de Odontopediatria, Faculdade de Odontologia de Ribeirão Preto, Universidade de São Paulo, Ribeirão Preto, São Paulo, Brazil; 3 Department of Microbiology and Immunology, Indiana University School of Medicine, Indianapolis, Indiana, United States of America; 4 Departamento de Ciências Biológicas, Faculdade de Ciências Farmacêuticas, Universidade Estadual Paulista “Júlio de Mesquita Filho”, Araraquara, São Paulo, Brazil; Instituto de Biofisica Carlos Chagas Filho, Universidade Federal do Rio de Janeiro, Brazil

## Abstract

The bioactive lipid mediator leukotriene B_4_ (LTB_4_) greatly enhances phagocyte antimicrobial functions against a myriad of pathogens. In murine histoplasmosis, inhibition of the LT-generating enzyme 5-lypoxigenase (5-LO) increases the susceptibility of the host to infection. In this study, we investigated whether murine resistance or susceptibility to *Histoplasma capsulatum* infection is associated with leukotriene production and an enhancement of *in vivo* and/or *in vitro* antimicrobial effector function. We show that susceptible C57BL/6 mice exhibit a higher fungal burden in the lung and spleen, increased mortality, lower expression levels of 5-LO and leukotriene B_4_ receptor 1 (BLT_1_) and decreased LTB_4_ production compared to the resistant 129/Sv mice. Moreover, we demonstrate that endogenous and exogenous LTs are required for the optimal phagocytosis of *H. capsulatum* by macrophages from both murine strains, although C57BL/6 macrophages are more sensitive to the effects of LTB_4_ than 129/Sv macrophages. Therefore, our results provide novel evidence that LTB_4_ production and BLT_1_ signaling are required for a histoplasmosis-resistant phenotype.

## Introduction

Histoplasmosis is caused by the dimorphic pathogenic fungus *Histoplasma capsulatum*, and infection occurs when microconidia are inhaled and transformed into yeast in the lung environment [1], [2]. Resistance to *H. capsulatum* infection requires cooperation between cells of the innate immune system, e.g., macrophages and dendritic cells, and cells of the adaptive immune system, e.g., CD4+ and CD8+ T cells. Interaction between the fungus and resident macrophages results in increased production of the proinflammatory cytokines IL-12, TNF-α, IL-1, IFN-γ and GM-CSF, which are essential for the development of a protective immune response [3], [4], [5]. Furthermore, production of leukotrienes (LTs), which are lipid mediators, is essential for the control of the host defense response during *H. capsulatum* infection [6], [7]. LTs are derived from the metabolism of arachidonic acid (AA) by the action of 5-lipoxygenase (5-LO) in association with 5-lipoxygenase-activating protein (FLAP). There are two major classes of LTs, namely LTB_4_ and cysteinyl-LTs (CysLTs), which include LTC_4_, LTD_4_ and LTE_4_ [8], [9], [10]; these two classes of mediators act by binding to the high-affinity G protein-coupled receptors BLT_1_ and CysLT_1_, respectively [11], [12]. LTB_4_ is predominantly synthesized by phagocytes in response to inflammatory or infectious stimuli and has a number of biological functions, including the stimulation of leukocyte migration, the activation of macrophages, eosinophils, neutrophils and T cells [13], [14], [15], opsonized and non-opsonized phagocytosis [7], [16], [17], [18], the production of antimicrobial mediators and microbial killing [19]. CysLTs elicit smooth muscle contraction, mucus secretion and edema during asthma and enhance microbial ingestion and killing [20]. Although both classes of LTs enhance macrophage antimicrobial killing, we have previously shown that LTB_4_ is approximately 10-fold more potent than CysLTs in enhancing the phagocytosis and killing of microbes [21]. The protective role of endogenous LTs has been demonstrated in several models of infection [6], [7], [18], [22], [23], [24], [25], [26], [27], and our group has a longstanding interest in investigating the role of LTs in pathogen infection, including histoplasmosis. Using pharmacologic and genetic approaches, we demonstrated that endogenous LTs augment the susceptibility of mice to primary and secondary *H. capsulatum* infections by amplifying fungal clearance and the production of the proinflammatory cytokines IL-12 and IFN-γ and the microbicidal molecule nitric oxide (NO). We have also shown that LTs are essential for enhancement of the activation and recruitment of effector and memory T cells to the site of infection [6], [7], [27].

The pattern of host resistance and susceptibility has been extensively studied in response to infection with *Paracoccidioides brasiliensis* [28], [29] and the protozoan parasite *Leishmania major* [25]. Although the production of proinflammatory cytokines is a crucial step in disease progression or resistance in most cases, whether lipids are also involved in these processes remains largely unknown. C57BL/6 mice infected with *H. capsulatum* are more susceptible to histoplasmosis than infected A/J mice [30], and spleen cells from susceptible C57BL/6 mice have been shown to produce lower amounts of IFN-γ than cells from resistant A/J animals. It has therefore been suggested that IFN-γ production is responsible for the differences observed in resistance/susceptibility during murine histoplasmosis. Nevertheless, because IFN-γ production and its effects can be dependent on LTB_4_ production as a model of toxoplasmosis, we speculated that LTB_4_ could be a missing link in the resistance to *H. capsulatum* infection. The data from our laboratory demonstrate that 129/Sv mice are more resistant to *H. capsulatum* infection compared to C57BL/6 mice [7], [27]. Therefore, we sought to investigate whether susceptibility and resistance to *H. capsulatum* infection are associated with differential LT production and/or action in these two animal strains.

## Results and Discussion

### C57BL/6 mice are more susceptible to *H. capsulatum* infection than 129/Sv mice

**Figure 1:**
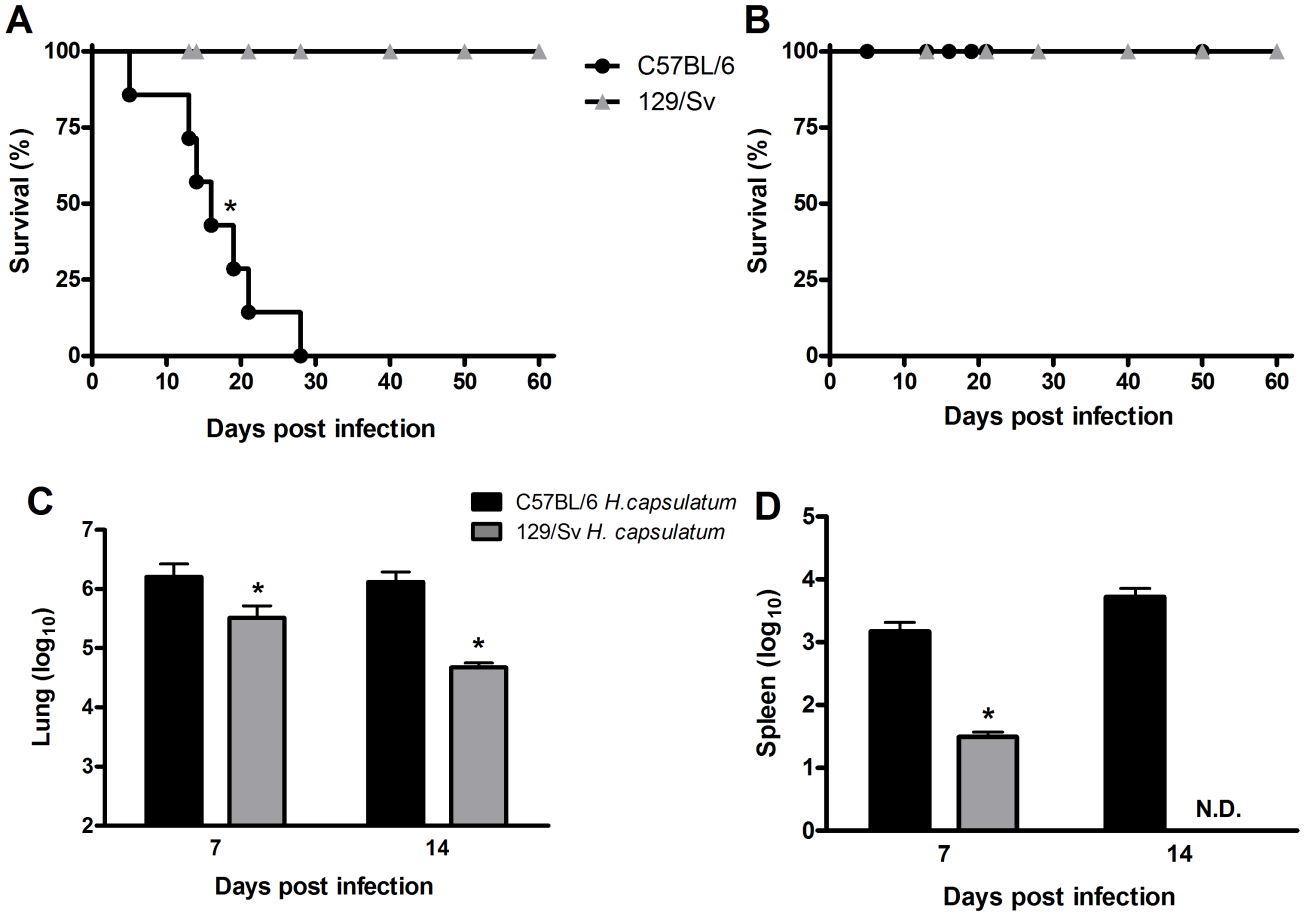
Survival rate and fungal burden in *H. capsulatum*-infected C57BL/6 and 129/Sv mice. C57BL/6 and sv129 mice were infected i.t. with 1×10^6^ (A) or 5×10^5^ (B) yeast cells, and their survival was followed for 60 days (n = 6). The fungal loads in the lungs (C) and spleen (D) of mice infected with 5×10^5^ yeast cells were evaluated at 7 and 14 days post-*H. capsulatum* infection. The data are expressed as the mean ± SEM from one representative experiment of a total of three experiments (n = 6/each experiment). *sv129 compared with C57BL/6. p<0.05 was considered significant.

We first confirmed the differences in the *H. capsulatum* infection susceptibility of C57BL/6 and 129/Sv mice. Both strains were infected with 1×10^6^ yeast cells; 100% of the 129/Sv mice survived up to 60 days after infection, whereas 100% of the C57BL/6 mice died during this period (Figure 1A). We then determined whether the increased mortality of the C57BL/6 mice correlated with impaired fungal clearance. We infected both strains of mice with a sub-lethal dose to evaluate the resolution of the infection. Although we observed a similar survival pattern (100%) in both strains throughout the study (Figure 1B), the fungal loads in the lungs and spleens of the C57BL/6 mice was significantly higher than those in the 129/Sv mice at 7 and 14 days post-infection (Figure 1C, D).

### C57BL/6 and 129/Sv mice exhibit distinct patterns of inflammatory cell recruitment to the lung

**Figure 2:**
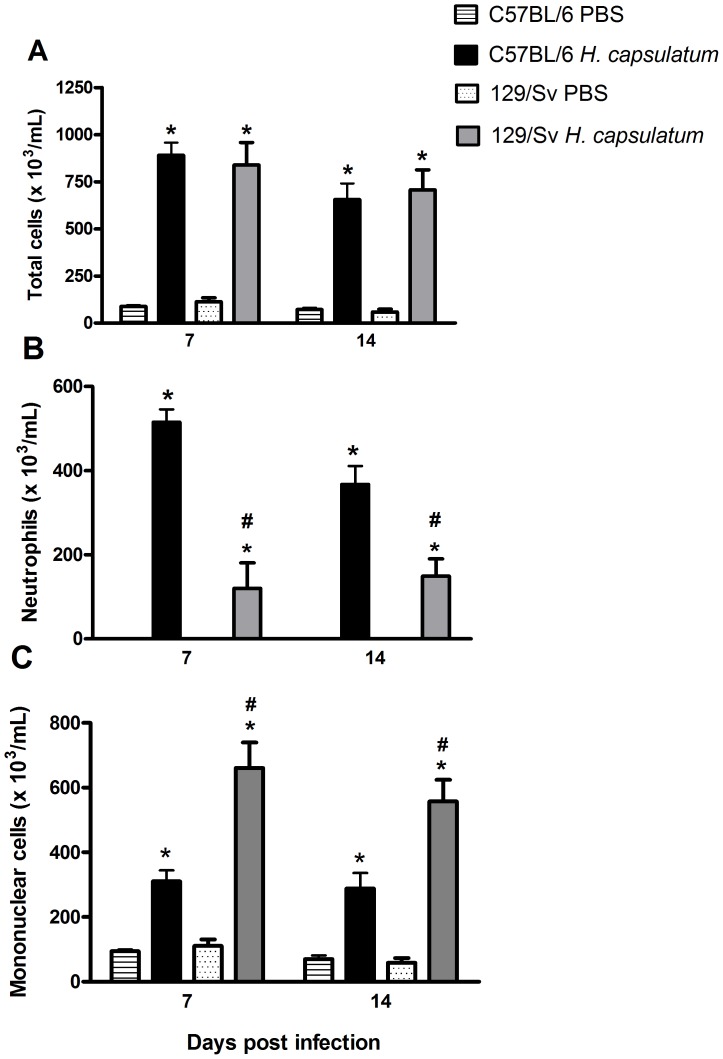
Differential leukocyte recruitment to the lung in resistant (129/Sv) and susceptible (C57BL/6) mice. Cells were obtained from mice at 7 and 14 days after i.t. injection of PBS or 5×10^5^ *H. capsulatum* yeast cells, as described in the Materials and Methods section. The total cells (A), neutrophils (B) and mononuclear cells (C) were enumerated and identified after Rosenfeld staining. The data are expressed as the mean ± SEM from one representative experiment of a total of three experiments (n = 6/each experiment). *C57BL/6 and sv129 compared with PBS; ^#^C57BL/6 compared with sv129. p<0.05 was considered significant.

**Figure 3:**
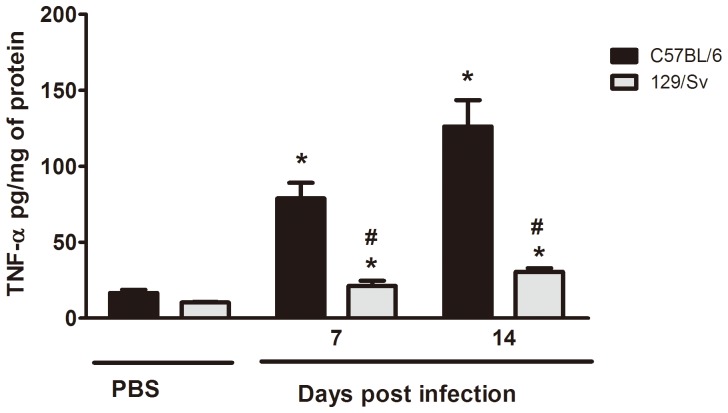
TNF-α production in lung of resistant (129/Sv) and susceptible (C57BL/6) mice. Lungs were removed at 7 and 14 days after i.t. injection of PBS or 5×10^5^ *H. capsulatum* yeast cells. TNF-α levels were determinate by ELISA. Data are presented as the mean ± SEM and are representative from one of two independent experiments (n = 6/each experiment). * 129/Sv compared with C57BL/6; ^#^129/Sv *H. capsulatum* compared with C57BL/6 *H. capsulatum*. p<0.05 vs. PBS.

We next sought to investigate whether lung leukocyte recruitment is associated with murine susceptibility and resistance. We observed higher neutrophil recruitment to the bronchoalveolar space in susceptible C57BL/6 mice compared with resistant 129/Sv mice at 7 and 14 days post-infection (Figure 2A). In contrast, we observed higher mononuclear cell recruitment to the bronchoalveolar space throughout the course of infection in resistant 129/Sv mice compared with susceptible C57BL/6 mice (Figure 2B). Moreover, the intense influx of neutrophils in C57BL/6 mice was associated with high levels of TNF-α (Figure 3). We have previously show both 5-LO deficient mice and mice treated with the MK886 and infected with *H. capsulatum* also exhibits high production of TNF-a which is associated with increased neutrophil migration to the lung [6], [31] Although our groups and others have previously shown that neutrophils are the predominant cell type in the early inflammatory response against *H. capsulatum* in the lung [1], [7], [15], [32], the role of these cells in the host defense against this mycosis remains unclear. *In vitro*, neutrophils exhibit potent microbicidal capacity against the mycelial form of *H. capsulatum* (25), whereas neutrophils exhibit only a microbiostatic effect against the yeast form *in vivo*. We have previously shown that the depletion of neutrophils by anti-Gr1 (RB6-8C5) antibody treatment restricts fungal growth in the early stages of infection and prevents the spread of yeast cells to other organs but does not modify the subsequent response [32]. In contrast, these results are in agreement with our previous results demonstrating that LT deficiency during *H. capsulatum* infection results in intense neutrophil recruitment and pulmonary injury, resulting in higher morbidity and mortality in mice [6], [7]. Increased neutrophil infiltration into the bronchoalveolar space after *Paracoccidioides brasiliensis* infection was also observed in susceptible B10.A mice compared with resistant A/J mice. In the referenced previous study, the antibody-mediated depletion of granulocytes in both strains decreased the survival of only the susceptible B10.A mice [33]. Although both mouse strains (B10.A and A/J) exhibited low neutrophil numbers associated with increased fungal recovery at the beginning of the infection, the spread of the fungus to other organs was observed in only the susceptible B10.A mice. We hypothesize that neutrophils may be important inflammatory cells involved in the control of fungal infection. However, granuloma formation in histoplasmosis is considered important for controlling the growth and dissemination of yeast and protecting against tissue injury [34]. Taken together, the persistence of neutrophil recruitment associated with decreased mononuclear cell recruitment into the lungs could be involved in the increased susceptibility of the C57BL/6 mice.

### The LTB_4_ synthetic capacity is higher in resistant mice compared to susceptible animals

**Figure 4:**
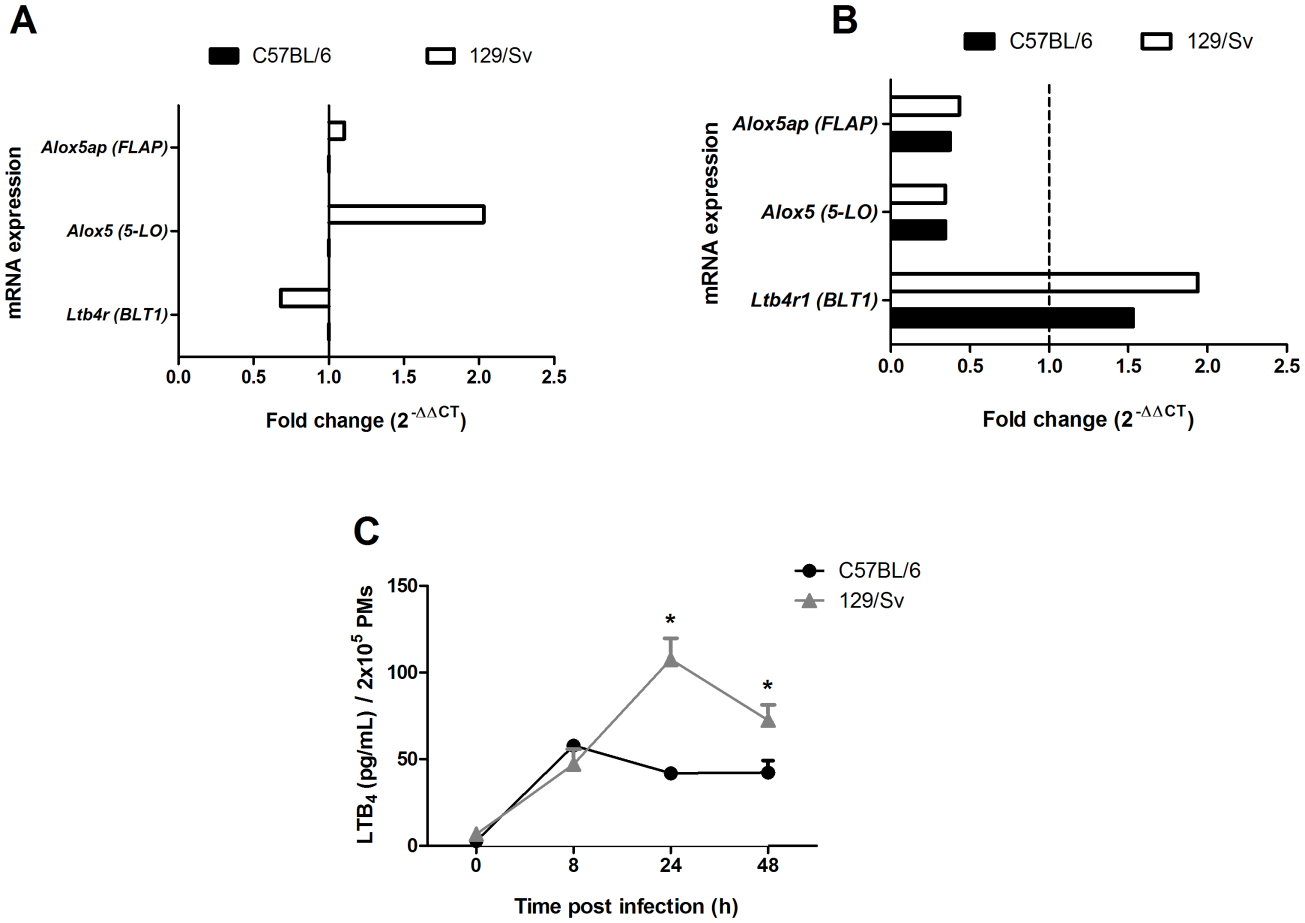
Differential 5-LO enzyme expression and LTB_4_ production in C57BL/6 and 129/Sv mice. The expression of *alox5*, *aloxp5* and *Ltbr1* mRNA in PMs from C57BL/6 and sv129 (A) and PMs infected with *H. capsulatum* (B) for 6 h as described in the Material and Methods. (B) LTB_4_ production by PMs from C57BL/6 and 129/Sv after *in vitro* infection with *H. capsulatum* (MOI = 1∶5) was measured by ELISA. The data are expressed as the mean ± SEM from one representative experiment of a total of two experiments (n = 3 to 5/each experiment). *sv129 compared with C57BL/6. p<0.05 was considered significant.

We previously demonstrated that LTs play a critical role in *H. capsulatum* infection by activating macrophage antimicrobial effector functions and recruiting T cells to the site of infection [6], [7], [27]. To evaluate whether 5-LO metabolites are involved in the susceptibility and resistance to *H. capsulatum* infection, we first analyzed the expression of the *alox5* (5-LO) gene and *alox5ap* (FLAP) expression using quantitative real-time PCR. We observed that the resistant mice exhibited higher levels of *alox5* (5-LO) gene expression but not *alox5ap* (FLAP) expression than PMs from the susceptible mice resident (Figure 4A). We did not observe any differences in the expression of *Alox5 and Alox5ap* mRNA in PMs from both strains infected with *H. capsulatum*. However, when PMs were infected with *H. capsulatum*, the expression of *Ltb4r* (BLT1) decreased ∼30% in PMs from resistant mice (Figure 4B). Moreover, 129/Sv PMs produced 157% (after 24 h) and 58% (after 48 h) more LTB_4_ than PMs from C57BL/6 mice upon *H. capsulatum* infection *in vitro* (Figure 4C). Higher LTB_4_ production in PMs from the resistant mice correlated with a marked increase in *alox5* (5-LO) mRNA expression but not *alox5ap* (FLAP) expression when compared with to the PMs from the susceptible mice resident (Figure 4B). Our results suggest that the genetic background of the host influences its ability to express 5-LO, which is reflected in disease susceptibility or resistance. We have previously shown that macrophages from susceptible BALB/c mice challenged with *Leishmania amazonensis* produce lower amounts of LTB_4_ than resistant C3H/HePas mice. Our study provides novel information regarding the differential production of LTB_4_ by different murine strains.

### The differential requirement of LTB_4_ enhances opsonized and non-opsonized phagocytosis in resistant mice

**Figure 5:**
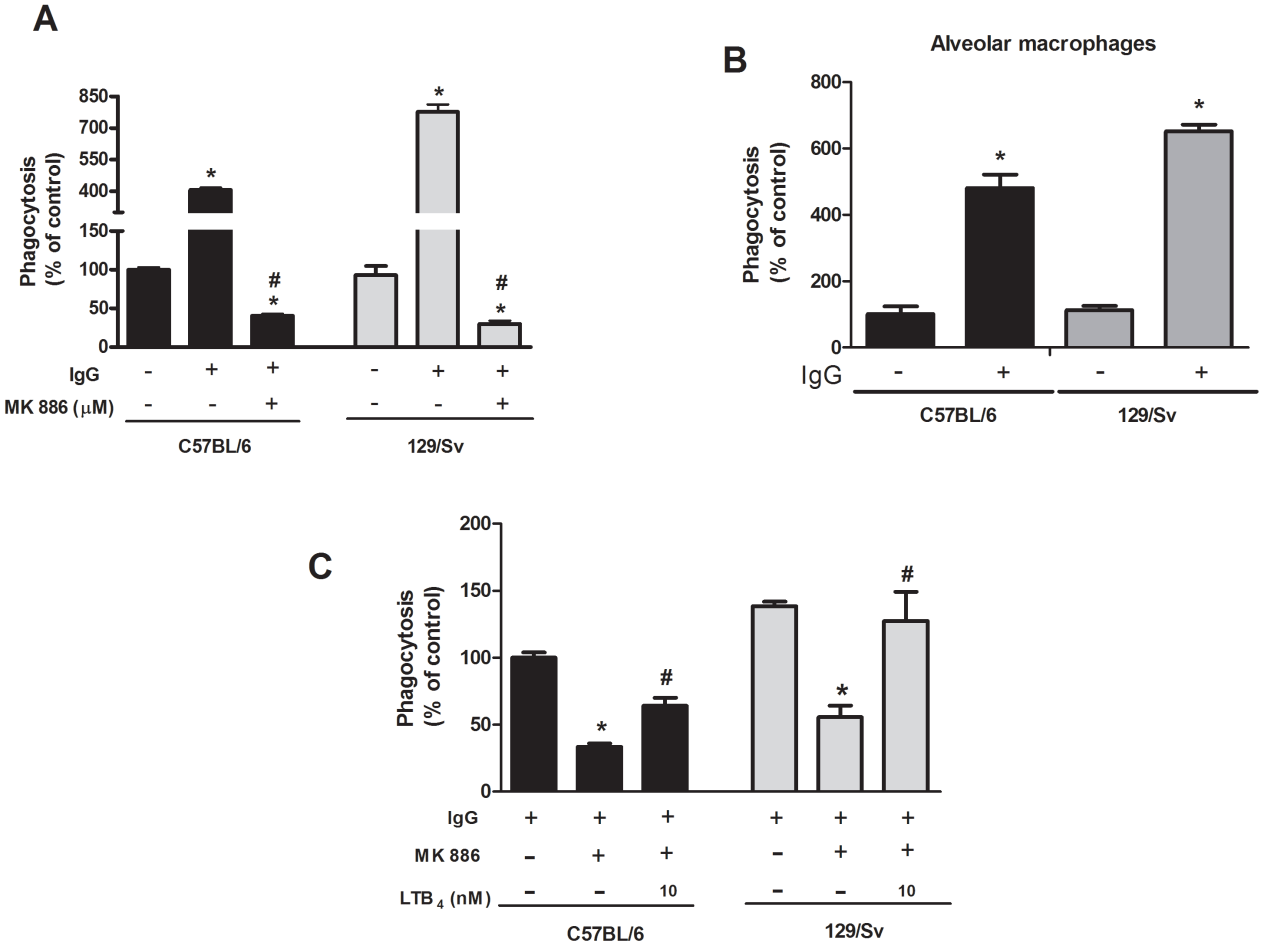
Effect of endogenous and exogenous LTB_4_ on the phagocytosis of *H. capsulatum* by C57BL/6 and 129/Sv macrophages. PMs from C57BL/6 and 129/Sv mice were incubated for 2 h with IgG-opsonized or non-opsonized yeast cells at a yeast-to-cell ratio of 1∶5. (A) PMs were pretreated with the LT synthesis inhibitor MK886 (1 µM) for 20 min. (B) AMs from C57BL/6 and 129/Sv mice were incubated for 2 h with IgG-opsonized or non-opsonized yeast cells at a yeast-to-cell ratio of 1∶5. (C) PMs were pretreated with the LT synthesis inhibitor MK886 (1 µM) for 20 min, followed by challenge with LTB_4_ (10 nm) for 5 min before the infection (C). The data are expressed as the mean ± SEM from one representative experiment of a total of two experiments (n = 3 to 5). *sv129 compared with C57BL/6. ^#^sv129 and C57BL/6 compared with MK886/LTB_4_ treatment. p<0.05 was considered significant.

We and other groups have demonstrated that LTB_4_ production is required for optimal macrophage effector mechanisms, such as phagocytosis and killing [7], [17], [18]. Because we observed that PMs from resistant mice produce higher amounts of LTB_4_ compared to PMs from susceptible mice, we sought to evaluate the importance of endogenous LTB_4_ in yeast ingestion by PMs from both mice strains. When PMs from resistant and susceptible mice were challenged with non-opsonized and IgG-opsonized yeast cells, no difference was observed in their abilities to phagocytose the non-opsonized yeast cells. However, as expected, coating the yeast cells with IgG increased the fungal uptake capacity of PMs from resistant mice by 50% compared with PMs from susceptible mice (Figure 5A). Similar phagocytic ingestion were observed in alveolar macrophages from resistant and susceptible mice (Figure 5B). Subsequently, we investigated whether the difference in the uptake of IgG-opsonized *H. capsulatum* by macrophages from susceptible and resistant mice could be explained by the LTB_4_/BLT_1_ signaling axis. The phagocytosis of opsonized yeast by macrophages from both strains was equally dependent on the endogenous synthesis of LTs because pretreatment of the cells with the FLAP inhibitor MK886 nearly abolished the uptake of the fungus by cells from both strains (Figure 5B). Interestingly, PMs from 129/Sv mice were more sensitive to MK886 compared to PMs from C57BL/6 mice, indicating a more profound LT dependency for ingestion in the resistant mice. Moreover, exogenous LTB_4_ partially restored phagocytosis of yeasts by PMs from susceptible mice whereas macrophages from resistant were total restored (Figure 5C).

**Figure 6:**
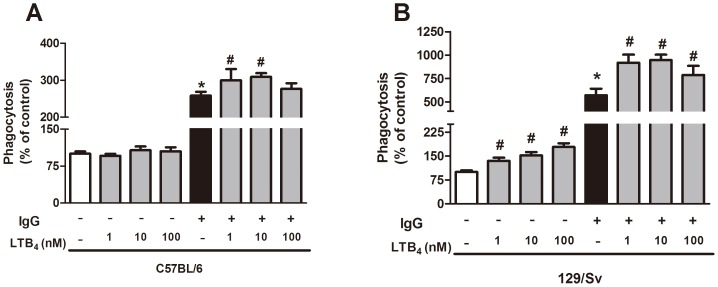
Effect of exogenous LTB_4_ on the phagocytosis of yeast by C57BL/6 and 129/Sv macrophages. PMs from C57BL/6 (A) and 129/Sv (B) mice were incubated for 2 h with IgG-opsonized or non-opsonized yeast at a yeast-to-cell ratio of 1∶5 in the presence or absence of exogenous LTB_4_. The data are expressed as the mean ± SEM from one representative experiment of a total of two experiments (n = 3 to 5). *sv129 compared with C57BL/6; ^#^129/Sv and C57BL/6 compared to LTB_4_ treatment. p<0.05 was considered significant.

We then investigated the effects of exogenous LTB_4_ on the phagocytosis capacity of both strains. Interestingly, LTB_4_ treatment enhanced *H. capsulatum* phagocytosis by PMs from 129/Sv mice. Compared with the opsonized fungus, LTB_4_ enhanced fungal ingestion by cells from both strains, but PMs from 129/Sv mice were more responsive to LTB_4_ than PMs from C57BL/6 mice (Figure 6A, B).

### *H. capsulatum* resistance is associated with a high level of BLT_1_ expression in the plasma membrane

**Figure 7:**
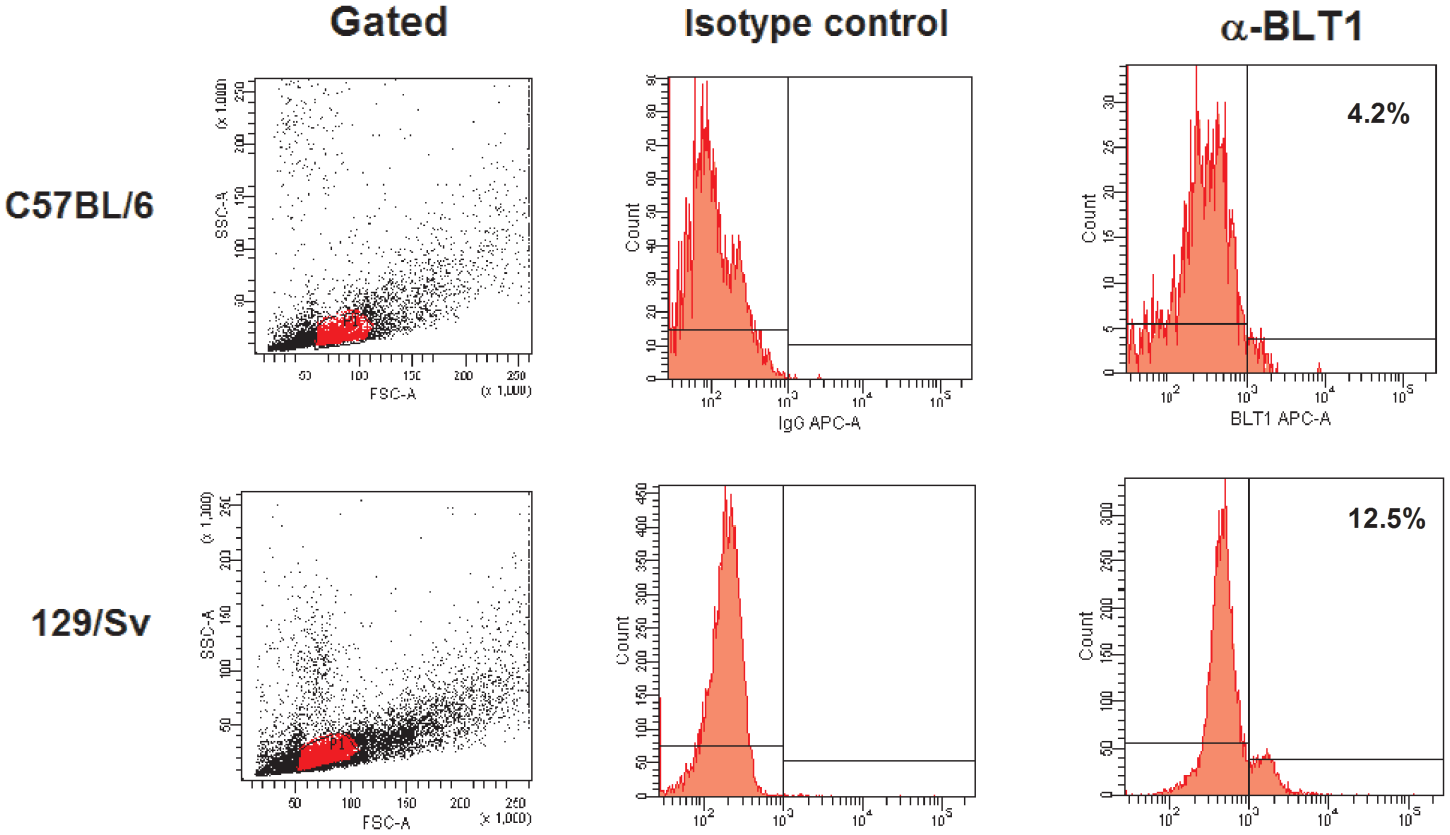
BLT_1_ expression on C57BL/6 (A) and 129/Sv (B) macrophages. The resident cells were obtained as described in the Material and Methods, and the expression of the higher affinity receptor for LTB_4_ was evaluated by flow cytometry. The mononuclear population was gated using the forward/side scatters and analyzed to determine the fluorescence intensity on the cells. The numbers in the histograms indicate the percentage of cells expressing the BLT_1_ receptor. The results shown are from one experiment and are representative of two independent experiments.

Because 129/Sv PMs are more sensitive to LTB_4_ than PMs from C57BL/6 mice, we assessed BLT_1_ expression in these mice. We observed that 12.5% of macrophages from resistant mice expressed the receptor, whereas only 4.2% of PMs from susceptible animals expressed the receptor (Figure 7). The roles of LTB_4_ and BLT_1_ in cell activation have been demonstrated by several investigators [17], [18], [35]. Talvani et al. [36] have shown that LTB_4_ increases NO production, phagocytosis and microbicidal activity in macrophages infected with *T. cruzi*. Furthermore, Serezani et al. [37] have demonstrated that LTB_4_/BLT_1_ signaling in macrophages is essential for activation of the MyD88/NF-κB pathway and the production of proinflammatory cytokines. The role of LTB_4_ in enhancing the phagocytosis of non-opsonized pathogens is controversial. However, Okamoto et al. [16] demonstrated that BLT_1_-deficient macrophages could ingest zymosan. Morato-Marques et al. [18] showed that LTB_4_ enhances the phagocytosis of *C. albicans*. Our results provide an answer for this discrepancy: whereas Okomoto et al. [16] used macrophages from C57BL/6 mice, which are less responsive to LTB_4_, we used macrophages from 129/Sv mice, which are highly responsive to LTB_4_. Moreover, Campos et al. [38] observed that the increased phagocytosis of IgG-opsonized targets by macrophages treated with LTB_4_ was dependent on the activation of signaling molecules, such as PKC-α/δ, ERK1/2 and PI3K, and that all of these events were dependent on the interaction between LTB_4_ and BLT_1_. Thus, we hypothesize that the decreased effect of LTB_4_ on yeast phagocytosis by PMs from susceptible mice may be related to a deficiency in BLT_1_ expression and, consequently, in the activation of the protein kinases involved in this process. In summary, our study further advances the knowledge in the field of murine histoplasmosis by dissecting the differential production of LTB_4_ and the subsequent responses in two mouse strains.

## Materials and Methods

### Animals

C57BL/6 and 129/Sv mice (6–8 weeks old) were obtained from The Jackson Laboratory (Bar Harbor, ME, USA) and were bred in the Faculdade de Ciências Farmacêuticas de Ribeirão Preto (Universidade de São Paulo, Brazil). All of the experiments were approved and conducted in accordance with the guidelines of the Animal Care Committee of Prefeitura of Campus of Ribeirão Preto (PCARP) of the University of São Paulo (Permit Number 08.1.390.53.3). Infected animals were maintained in biohazard facilities and housed in cages within a laminar flow safety enclosure under standard conditions.

### Preparation of *H. capsulatum* and infection

*H. capsulatum* was recovered from a patient at the Hospital das Clínicas at the Faculdade de Medicina de Ribeirão Preto of the Universidade de São Paulo, Brazil. Yeast cells were obtained by fungal culture at 37°C on BHI blood agar for 7–10 days and used once their viability reached ≥90%, as measured using fluorescein diacetate and ethidium bromide. The infection was performed as described previously [7]. Briefly, mice were anesthetized with ketamine (100 mg/kg) and xylazine (10 mg/kg), restrained on a small board and intratracheally (i.t.) infected with 5×10^5^ or 1×10^6^ viable *H. capsulatum* yeast cells in 100 µl of PBS. The control animals received 100 µl of phosphate-buffered saline (PBS) i.t.

### Culture of organ-infecting *H. capsulatum*

The recovery of *H. capsulatum* from the lungs and spleens of the infected mice was performed as previously described by Secatto et al. [7]. Three serial dilutions were performed, and 0.2 ml each serial-dilution was plated on a BHI-agar-blood agar. The number of yeast cells was counted after incubation at 37°C for 21 days and used to express the fungal burden in the tissues. The results are expressed as the number of colony forming units (CFUs) per lung and spleen.

### Bronchoalveolar lavage fluid (BALF) analysis and differential cell counts

On days 7 and 14 after infection, the animals were euthanized in a carbon dioxide chamber. The cells present in the bronchoalveolar space were enumerated by differentially counting the neutrophils and mononuclear cells in the BALF, as previously described [6]. The numbers of neutrophils and mononuclear cells were determined using a Neubauer chamber, and the cells were identified by Cytospin and panoptic staining [15].

### Measurement of TNF-α and total protein

Lungs were removed on days 7 and 14 post-infection to measure TNF-α. Briefly, tissue was homogenized (Mixer Homogenizer, Labortechnik, Germany) in 2 ml of RPMI 1640, centrifuged and stored at −70°C until assayd. A specific enzyme immunoassay was used according to the manufacturer's instructions (R&D Systems, Minneapolis, MN). The sensitivity of the assay was <10 ng/mL.

Concentration total proteins were quantified in the lungs homogenate of mice by Coomassie protein assay reagent (Rockford, USA), according to the manufacturer's instructions. Data was expressed concentration of TNF-α (pg/ml)/total protein (mg/ml).

### Isolation of peritoneal macrophages (PMs) and alveolar macrophages (AMs) and cell culture

PMs were obtained from naive C57BL/6 and 129/Sv mice, as described previously [7]. AMs were obtained from naive C57BL/6 and 129/Sv mice by lung lavage as described previously [39]. The percentage of macrophages was determined microscopically with a panoptic stain, and the purity of the cells was higher than 95%. Cells were cultured overnight in RPMI containing 10% fetal bovine serum and 1% gentamicin. After 24 h, the cells were washed twice with warm serum-free medium to remove all of the non-adherent cells, and the adherent cells were used to assess the degree of fungal phagocytosis.

### Measurement of LTB_4_

LTB_4_ production was measured in the supernatant of the macrophage culture according to the manufacturer's instructions (Cayman Chemical, Ann Arbor, Michigan, USA).

### Phagocytosis assays

The degree of yeast phagocytosis was assessed using the protocol described by Secatto et al. [7]. Briefly, *H. capsulatum* yeast cells were labeled with FITC (Amresco,Solon,OH) for 1 h at 37°C, as described by Serezani et al. [40]. The FITC-labeled *H. capsulatum* cells were further diluted in RPMI and incubated with PMs at a ratio of 1∶5 (yeast cells∶macrophages). After 2 h of incubation in the dark (37°C, 5% CO_2_), uningested yeast cells were removed by washing with PBS, and the residual extracellular FITC was quenched with trypan blue (250 mg/ml; Molecular Probes) for 1 min. Fluorescence was determined using a microplate fluorometer (485 nm excitation/535 nm emission, SPECTRAFluor Plus; Tecan, Research Triangle Park, NC). In some experiments, PMs were pretreated for 5 min with LTB_4_ (Cayman Chemical, Ann Arbor, Michigan) or MK888 (FLAP inhibitor; Merck Frosst, Canada) diluted in RPMI prior to being co-cultured with FITC-labeled *H. capsulatum* cells.

### Flow cytometric analysis

PMs from control C57BL/6 and 129/Sv mice were obtained as decribed above and adjusted to concentration of 1×10^6^ cells/100 µL. FcγRs were blocked by addition of unlabeled anti-CD16/32 for 40 min. After that, the cells were incubated with an anti-BLT1 mAb (Alexa647) (AbD Serotec) and isotype controls for 30 min at 4°C. The gate was perfomed using SSC/FSC parameters in FACSCanto (BD Biosciences) and FACS Diva software.

### RNA isolation and quantitative real-time PCR (qRT-PCR)

The expression of mRNA was evaluated by qRT-PCR after 6 h of infection cells with *H.capsulatum* or *medium*. mRNA was obtained using an RNeasy Mini Kit (Qiagen) according to the manufacturer's instructions. The levels of *alox5ap*, *alox5* and *Blt1* mRNA were normalized to the mRNA levels of *Actb* and *Gapdh using* RT^2^ Profiler PCR Array kit (Qiagen). The results were analyzed using the 2^−ΔΔCt^ method, and the level of gene expression in C57BL/6 cells was set at 1.

### Statistical analysis

The data are presented as the mean ± SEM. Comparisons were performed using ANOVA, followed by the Newman-Keuls test. All of the statistical analyses were performed using the Prism 5.0 statistical program (GraphPad Software, San Diego, CA, USA). Any differences in survival were analyzed using the log-rank test. Differences with a p-value of less than 0.05 were considered statistically significant.
